# Enhanced Proton Conductivity and Methanol Permeability Reduction via Sodium Alginate Electrolyte-Sulfonated Graphene Oxide Bio-membrane

**DOI:** 10.1186/s11671-018-2493-6

**Published:** 2018-03-13

**Authors:** N. Shaari, S. K. Kamarudin, S. Basri, L. K. Shyuan, M. S. Masdar, D. Nordin

**Affiliations:** 1 Fuel Cell Institute, Universiti Kebangsaan Malaysia (UKM), 43600 Bangi, Selangor Malaysia; 2 Department of Chemical and Process Engineering, Faculty Of Engineering and Built Environment, Universiti Kebangsaan Malaysia (UKM), 43600 Bangi, Selangor Malaysia

**Keywords:** Bio-membrane, Sodium alginate, Sulfonated graphene oxide, DMFC

## Abstract

The high methanol crossover and high cost of Nafion® membrane are the major challenges for direct methanol fuel cell application. With the aim of solving these problems, a non-Nafion polymer electrolyte membrane with low methanol permeability and high proton conductivity based on the sodium alginate (SA) polymer as the matrix and sulfonated graphene oxide (SGO) as an inorganic filler (0.02-0.2 wt%) was prepared by a simple solution casting technique. The strong electrostatic attraction between -SO_3_H of SGO and the sodium alginate polymer increased the mechanical stability, optimized the water absorption and thus inhibited the methanol crossover in the membrane. The optimum properties and performances were presented by the SA/SGO membrane with a loading of 0.2 wt% SGO, which gave a proton conductivity of 13.2 × 10^−3^ Scm^−1^, and the methanol permeability was 1.535 × 10^−7^ cm^2^ s^−1^ at 25 °C, far below that of Nafion (25.1 × 10^−7^ cm^2^ s^−1^) at 25 °C. The mechanical properties of the sodium alginate polymer in terms of tensile strength and elongation at break were improved by the addition of SGO.

## Background

The simple conversion of chemical energy from a fuel through a chemical reaction into electricity can only be done by a fuel cell device. Regarding this capability, the direct methanol fuel cell (DMFC) has received great attention because it can operate using only 17% methanol as the fuel to produce electricity with reduced pollutant emissions compared with other methods and is also safe to use while flying [1]. DMFC has wide capabilities in many applications, such as medical tools, hearing aids, and portable tools. Unfortunately, its application has been hindered due to its lack of commercialization, which is attributed to issues such as the high cost of production (approximately 1000 USD m^−2^) [2], high methanol permeability of commercialized membranes (Nafion) and low reactivity and low durability of the current electrocatalysts (palladium and ruthenium) [3]. The proton electrolyte membrane is the most vital component in DMFC because it functions as a fuel and oxidant separator, as well as a path for conducting protons; consequently, it can have a substantial effect on the overall system efficiency. Among the required membrane characteristics, the membrane should have high proton conductivity and the ability to effectively block the methanol from crossing the membrane to avoid cathode side poisoning [4]. In addition, it is important to ensure the use of non-hazardous, inexpensive raw materials for the membrane. The current commercial membrane (Nafion) does not meet these major requirements; therefore, it is not a good membrane for DMFC applications due to its high methanol permeability, high cost, and use of hazardous materials. In addition, its proton conductivity is affected by these problems, consequently limiting its effectiveness in DMFC applications. Currently, biomaterials are receiving attention because they are safe and environmentally friendly, classifying them as green technology materials. As a new and excellent biomaterial, alginates have intrigued many researchers from various areas for applications including tissue engineering, biomedicine, delivery vehicles for drugs, food packaging, and DMFC [5]. Alginate is a prominent water-soluble polysaccharide found in brown seaweed, and it consists of (1-4)-linked β-d-mannuronic acid (M) and α-l-guluronic acid (G) units. It has very high water absorption and can absorb 200–300 times its own weight in water [6]. The proton conduction ability of pristine alginate is low due to the absence of continuous transfer pathways and the weak conducting ability of the polymer [6–9]. Previous studies showed that the most effective method to enhance the mechanical properties and specialize the other properties of this polymeric material is to introduce an inorganic material and a polymer backbone [7]. Composite materials can extend or provide novel capabilities that are difficult to obtain by using each component individually. For instance, the mechanical strength of alginate has been successfully enhanced by introducing carbon nanotube and graphene oxide into the alginate polymer matrix [3, 10, 11]. Previous studies on the development of biopolymer-based membranes have shown good potential when combined with other materials such as inorganic or synthetic polymers, e.g., double layer-chitosan (1.67 × 10^−6^ cm^2^ s^−1^) [12], chitosan-PVA/Nafion (2.2 × 10^−6^ cm^2^ s^−1^) [13], chitosan-SHNT (0.76 × 10^−2^ Scm^−1^) [14], chitosan-zeolite (2.58 × 10^−2^ S cm^−1^) [15], chitosan-PMA (1.5 × 10^−2^ S cm^−1^) [16], chitosan-sodium alginate (4.2 × 10^−2^ S cm^−1^) [17], alginate-carrageenan (3.16 × 10^−2^ S cm^−1^) [18], sulfonated chitosan-SGO (72 × 10^−2^ S cm^−1^) [19], PVA-sodium alginate (9.1 × 10^−2^ S cm^−1^) [20], biocellulose-Nafion (7.1 × 10^−2^ S cm^−1^) [21], chitosan-SPSF (4.6 × 10^−2^ S cm^−1^) [22], chitosan-silica/carbon nanotube (CNT) (2.5 × 10^−2^ S cm^−1^), chitosan-PVP (2.4 × 10^−2^ S cm^−1^) [23], nanocellulose/polypyrrole (1.6 mW cm^−2^) for enzymatic fuel cell [24], cellulose nanofibres (CNFs) (0.05 × 10^−3^ S cm^−1^) and cellulose nanocrystals (CNCs) (4.6 × 10^−3^ S cm^−1^) [25], bacterial cellulose (BC)/poly (4-styrene sulfonic acid) (PSSA) (0.2 S cm^−1^) [26], and imidazole-doped nanocrystalline cellulose (2.79 × 10^−2^ S cm^−1^) [27]. However, the number of biopolymer-based membranes developed is too small compared to the studies involving synthetic polymers in many areas including fuel cells. Additionally, it is undeniable that chitosan has received more attention than the other carbohydrate polymers.

Graphene oxide is a promising carbon-based material with high potential in many applications, including electronics, nanocomposites, biomedicine, and fuel cells. Graphene oxide has excellent properties, such as a high aspect ratio, high conductivity, high mechanical strength, unique graphitized plane structure, and electrical insulating properties [28]. As an additive material in a hydrophilic polymer matrix, it provides high resilience to resist swelling caused by moisture. Furthermore, graphene oxide would be preferable to CNT due to its much lower cost, which makes it the most suitable candidate for membranes in DMFC applications [29]. Previous studies showed that GO strengthened natural polymers such as chitosan films and chitosan-gelatin porous monoliths [19, 30]. Bayer et al. [31] prepared a GO paper, which showed hydrogen permeability three times lower than Nafion and proton conductivity of 49.9 mScm^−1^ using an in-plane technique. The direct liquid fuel cell (DLFC) performance was excellent when Lue et al. [32] introduced GO into Nafion. However, the performance of GO as a proton conductor is limited because it lacks functional groups that can be proton carriers in the membrane, which adversely affects proton conductivity and decreases fuel cell performance [19]. Karim et al. [33] reported that the conductivity of the GO nanosheet in their study was 15 mS cm^−1^ and the GO conductivities reported by Hatakeyama et al. [34] and Bayer et al. [35] were 0.4 mScm^−1^ and 0.55 mScm^−1^, respectively. Based on these weaknesses, sulfonated GO is considered as a better option than GO for this application because sulfonated GO has shown increased proton conductivity, and it facilitates the formation of a homogeneous membrane due to the high compatibility between the GOS and SO_3_H [19]. Keith et al. [36] presented a SGO paper that showed a high maximum power density of 113 mWcm^−2^ at 0.39 V for polymer electrolyte membrane fuel cell (PEMFC). The advantages of –SO_3_H incorporation are as follows: (i) the acid groups can offer supplementary hopping sites for proton movement, and (ii) the electrostatic attractions will improve the thermal and mechanical stabilities by interfering with the alginate chain mobility and packing. Based on our research, no nanocomposite alginate/SGO material has been produced yet using this method. The use of biomaterials in the application of electrical devices will lead to interdisciplinary research between the biological sciences and sustainable energy technologies. Therefore, this research will combine the advantages of alginate and SGO to form a novel bio-membrane with high durability, good proton conductivity, and methanol permeability with the goal that it will perform better than Nafion or other commercial proton exchange membranes (PEMs) as well as being much cheaper to produce than Nafion.

## Methods

### Materials

TIMREX PG25 natural graphite was purchased from TIMCAL Ltd. Concentrated sulfuric acid (H_2_SO_4_, 95%), methanol (CH_3_OH, 99.7%), potassium permanganate, hydrochloric acid, hydrogen peroxide aqueous solution (H_2_O_2_, 35%), calcium chloride, ethanol, sulfanilic acid, sodium nitrite solution, and glycerol were obtained from Sigma Aldrich. These chemicals were used as received without further purification. Deionized (DI) water through a Millipore system (Milli-Q) was used in all experiments.

### Membrane Preparation

Hummer’s method was modified and applied to provide a GOS from natural graphite [10, 37]. First, 2 g of graphite was mixed with 150 ml of H_2_SO_4_ (95%) in a 500-ml flask. The mixture was stirred for 30 min in an ice bath. Under continuous and vigorous stirring, 15 g of potassium permanganate was added to the mixture. The addition rate was carefully controlled to maintain the reaction temperature at 20 °C. The mixture was then stirred and left overnight at room temperature, followed by the addition of 180 ml of water under vigorous stirring and reflux at 98 °C for 24 h; this caused the solution to turn a yellow color. Eighty milliliters of 35% H_2_O_2_ was added to the reaction mixture, which was allowed to cool to room temperature in order to quench the reaction with KMnO_4_. The resulting GO was washed by rinsing with 5% HCl followed by centrifugation. Finally, the product was rinsed with DI water several times, filtered and dried under vacuum conditions.

**Scheme 1:**
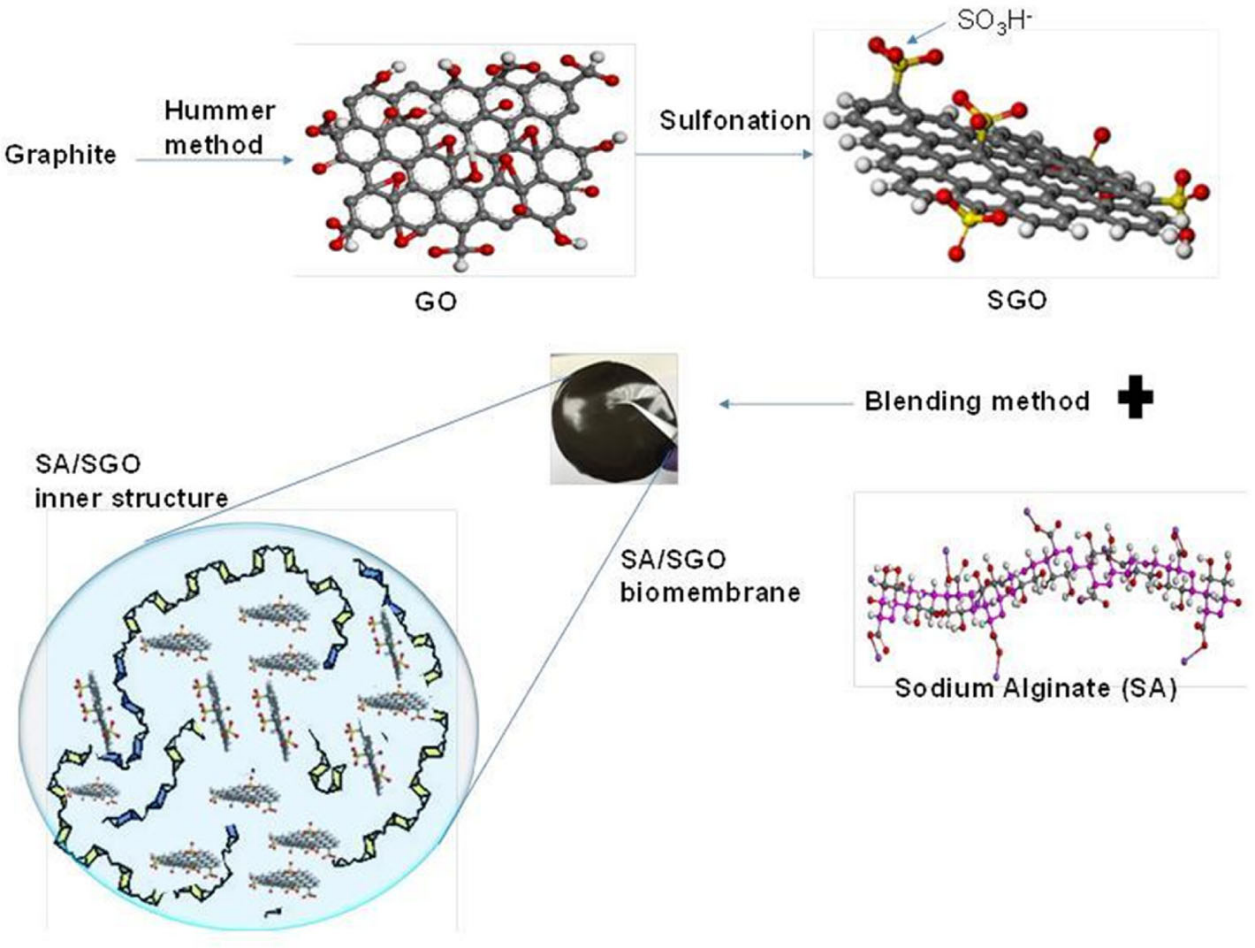
Sulfonated graphene oxide (SGO) filler and SA/SGO biomembrane preparation method

Fifty milliliters of graphene oxide was added to 8 ml of a 0.06 M sulfanilic acid solution at 70 °C. With continuous stirring, 2 ml of sodium nitrite solution was added dropwise to the mixture and allowed to stand for 12 h at a constant temperature of 70 °C. After the reaction was complete, the mixture was washed and collected by centrifugation. The collected SGO was washed several more times with water until reaching pH 7. The SGO particles were characterized by X-ray photoelectron spectroscopy (XPS). Sodium alginate was dissolved in 1% (*w*/*v*) double-distilled water to obtain a solution of alginate. The SGO content added to the alginate solution varied, with values of 0.02, 0.05, 0.09, 0.13, 0.17, and 0.2 wt% to produce a composite film. The mixture was stirred continuously for 60 min with a magnetic stirrer. The heterogeneous solution was transferred to a glass substrate and was left at 60 °C for 72 h to allow for the thin film formation process. The dried alginate/sulfonated graphene oxide membrane was then crosslinked using a calcium chloride/glycerol solution to increase the mechanical strength and to reduce the hydrophilic properties of alginate. The membrane was immersed for 30 min in 100 ml of crosslinking solution whose cation concentration was maintained at 1.5% *w*/*v*. Finally, any free cations were removed from the membrane surface by washing with DI water, and the membrane was dried at 25 °C. The preparation method is summarized in Scheme 1.

### Membrane Characterization

The Fourier transform infrared (FTIR PERKIN ELMER) spectra of graphene oxide, sulfonated graphene oxide, and the membrane were analyzed. The FTIR wavelength was in the range of 4000–500 cm^−1^. The microstructure of the film membranes was examined using a field emission scanning electron microscope (FEI QUANTA 400 FESEM) with an operating voltage of 5 kV as a precaution for the bio-material-based sample. The high-resolution transmission electron microscopy (HRTEM) analysis was carried out using o Digital TEM HT7700 operated at an accelerating potential of 300 kV.

Samples were prepared on grids with lacey carbon support film. XPS was used to determine the chemical composition of the sample surface using an Axis Ultra DLD. The mechanical strength of the SA/SGO membrane was tested with a Universal Testing Machine, including tensile strength, Young’s modulus, and elongation at break. The load used was 3 kN at room temperature. Changes in the weight and length (or thickness) of wet and dry membranes can determine the rate of water absorption and the swelling ratio of the membrane. Typically, the membrane was soaked in water for 2 days at 30 °C. For the wet membrane, the weight and length were recorded, and then, the water in the membrane and the liquid droplets on the surface of the membrane were removed. In addition, the moist membrane was dried under vacuum pressure and temperature of 120 °C for at least 24 h. The weight and length of the membrane in the dry state were also recorded. Using Eqs. 1 and 2, water intake (WU%) and swelling ratio (SW%) can be determined, where *L*_wet_ represents the wet mass and *L*_dry_ represents the dry mass obtained from the length of wet and dry membranes, respectively.

1 $$ \mathrm{WU}\%=\frac{{\mathrm{mass}}_{\mathrm{wet}}-{\mathrm{mass}}_{\mathrm{dry}}}{{\mathrm{mass}}_{\mathrm{dry}}}\times 100 $$

2 $$ \mathrm{SW}\%=\frac{L_{\mathrm{wet}}-{L}_{\mathrm{dry}}}{L_{\mathrm{dry}}}\times 100 $$

The methanol uptake calculation is the same as the water uptake calculation, except that the solution for immersion is changed to methanol rather than DI water.

The proton conductivity of the prepared membrane was calculated using a four-electrode conductivity cell connected to a potentiostat/galvanostat (WonATech) operating over a frequency range of 1 MHz down to 50 Hz. The membranes (1 cm × 4 cm in size) must be soaked in water for 24 h for the conductivity readings under the fully hydrated state. The potentiostat was run to obtain the graph of voltage versus current. The gradient of the straight line is the membrane resistance. Scheme 1 presents the cell of the proton conductivity test. The proton conductivity can be calculated using the following formula:

3 $$ \sigma =\frac{L}{RWT} $$

where *L* is the distance between the two electrodes, *W* is the width of the membrane, *T* is the membrane thickness, and *R* is the resistance of the membrane, similar to the method in previous works [38, 39].

Two tank liquid permeability cells with 20 *v*/*v*% methanol were used to determine the methanol permeability of the membrane. The differences in the concentration of methanol result in methanol crossover through the membrane, and methanol permeability can be determined. Equation 3 is used to calculate the permeability of methanol:

4 $$ P=\frac{1}{Ca}\left(\frac{\Delta  Cb(t)}{\Delta  t}\right)\left(\frac{LVb}{A}\right) $$

where *P* is the membrane diffusion permeability for methanol (cm^2^ s^−1^), C_a_ is the methanol concentration in the feed chamber, i.e., cell A (mol L^−1^), *∆Cb*(*t*)/*∆t* is the methanol molar concentration variation in cell B as a function of time (mol L^−1^ s), *V*_b_ is the volume of each diffusion reservoir (cm^3^), *A* is the membrane area, and *L* is the membrane thickness (cm).

The membrane characteristics can be determined by calculating the selectivity of the membrane, which can be achieved by high proton conductivity and low methanol permeability. The formula used for calculating the selectivity is as follows:

5 $$ \varphi =\frac{\sigma }{P} $$

where *φ* represents selectivity, *σ* represents ionic conductivity, and *P* represents methanol permeability.

## Results and Discussion

### Characterization of Sulfonated Graphene Oxide (SGO) and SA/SGO Biomembrane

**Fig. 1:**
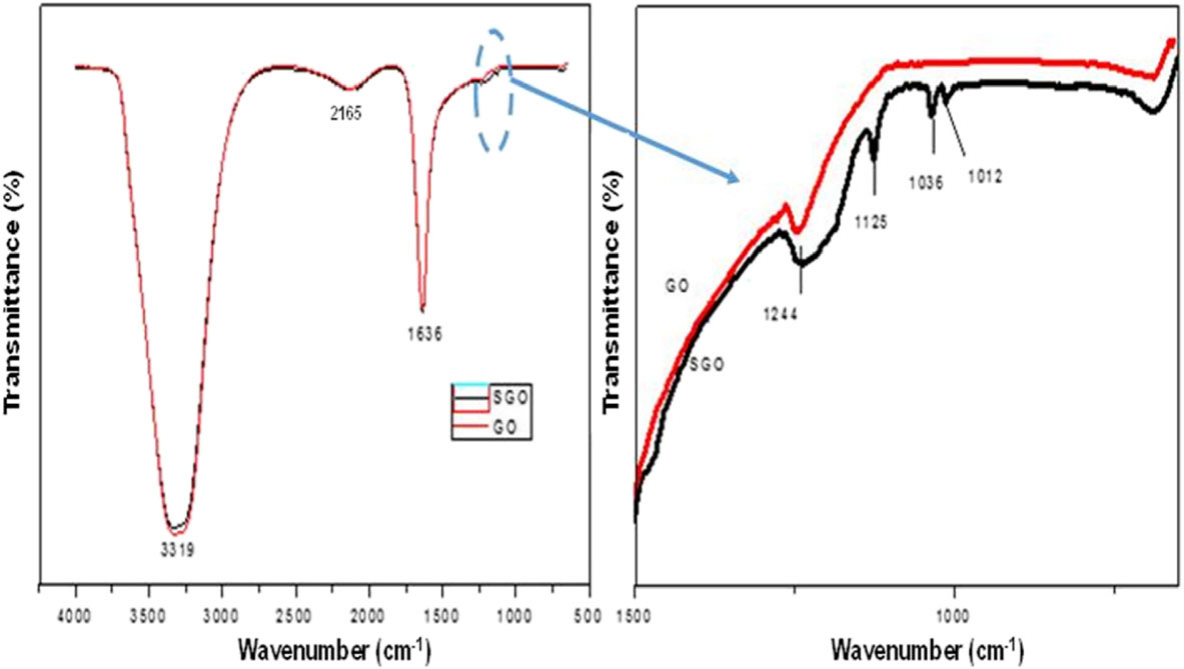
**a**, **b** FTIR spectra for graphene oxide (GO) and sulfonated graphene oxide (SGO)

The FTIR spectra in Fig. 1a, b show the difference between GO and SGO, which can be clearly observed. Figure 1b is the magnification of Fig. 1a to obtain a clearer view of the peaks in the SGO spectra. The spectrum of SGO shows a new band at 1244 cm^−1^, which is the typical absorbance of a sulfonic acid group (-SO_3_H), whereas the GO spectrum does not contain this band [40]. In addition, the spectrum shows new peaks at the wavelengths of 1012, 1036, and 1125 cm^−1^, which are considered to be the symmetric and asymmetric stretching vibrations of SO_3_H^−^ groups. This new spectrum reveals that the graphene oxide solution was successfully modified into sulfonated graphene oxide using the simple method described above. At the same time, the sulfonation modification still kept the functional groups in GO such as the hydroxyl group at 3319 cm^−1^ and the carboxyl group at 1636 cm^−1^. Further confirmation of the presence of SO_3_H^−^ groups can be determined by XPS analysis.

**Fig. 2:**
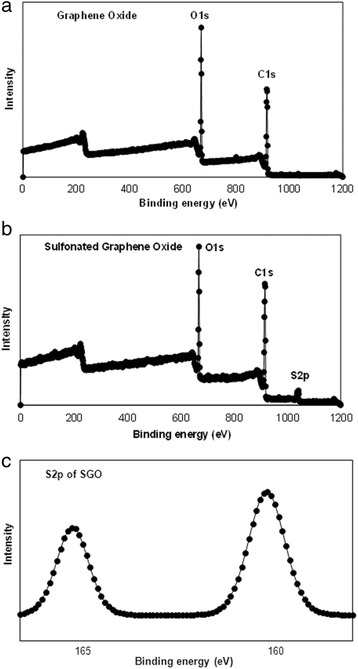
XPS of **a**, **b** wide spectra GO and SGO and **c** S2p spectra of SGO

Figure 2 shows the XPS spectra of the GO and SGO membranes in which the scanning spectra are in the range of 0–800 eV to recognize the surface of the existing elements via a measurable analysis. It can be observed that the C1s and O1s signals appeared at 286 and 531 eV, respectively, in both the GO and SGO spectra. It is also noticed that after the sulfonic acid groups were introduced into GO, a new S2p peak appeared at 168 eV. Sulfonic groups in SGO contributed to a slightly increased intensity in the O1s spectra compared with that of GO. The high-resolution spectrum of C1s, which is referred to as Gaussian spectral deconvolution, confirmed that GO was successfully customized via chemical modification [41]. The figure inside Fig. 2b is the S2p spectra for functionalized GO at a larger magnification. The binding energy of the sulfonic groups contributed to the appearance of the S2p peak at 168 eV, and this peak confirmed that sulfonic acid groups were successfully attached to the GO nanosheet backbone [41, 42].

**Fig. 3:**
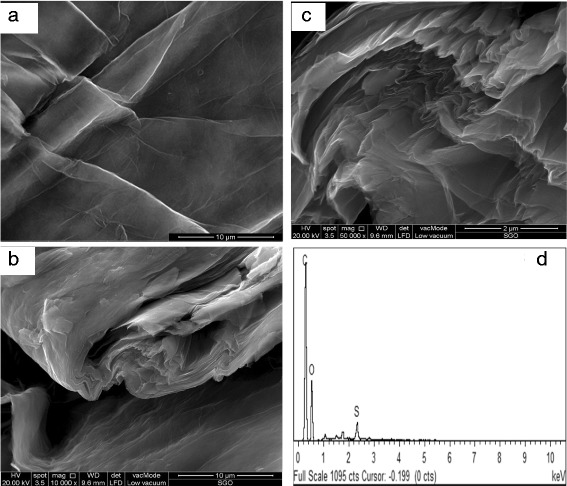
**a** FESEM image of GO. **b**, **c** FESEM images of SGO with various magnification and **d** EDX of SGO

The successful production of GO via the Hummer’s method was confirmed by the sheet-shaped GO morphology as shown in the FESEM image (Fig. 3a). Bai et al. [43] also generated GO with Hummer’s method. The results of their studies showed that the morphologies of both GO and RGO appeared to be slightly folded and formed some wrinkles, which resemble the GO morphology in this study.

The FESEM image of SGO in Fig. 3b, c has a crumpled and rougher surface compared with the surface of GO, which is most likely due to the effects of the sulfonation process, confirming that the modification method was also successfully applied [41, 44]. This correlates with the existence of a new peak in the FTIR transmittance spectra, which belongs to the sulfonic group. Moreover, the presence of sulfonic groups was also confirmed in the GO sheet via the XPS analysis. SGO was different from GO, which had a multi-layered structure without any aggregation. The applied modification method leads to the formation of a layered and restacked structured; thus, SGO demonstrated its flexibility. The energy dispersive X-ray (EDX) result presents that 1.76 wt% of sulfur element exists in the SGO sheets (Fig. 3d).

**Fig. 4:**
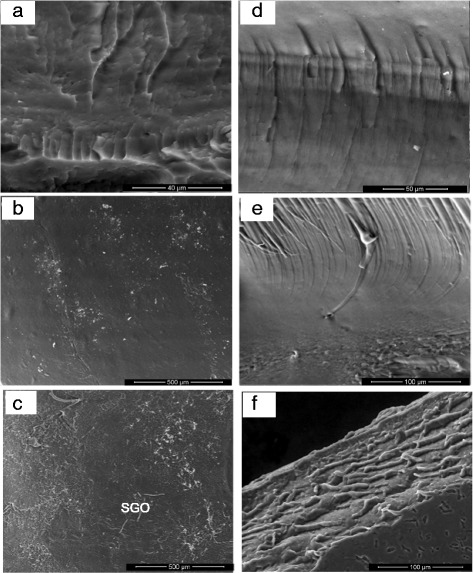
FESEM images of surface morphology and cross-section for **a**, **d** sodium alginate, **b**, **e** SA/SGO4, and **c**, **f** SA/SGO6 biomembranes

**Fig. 5:**
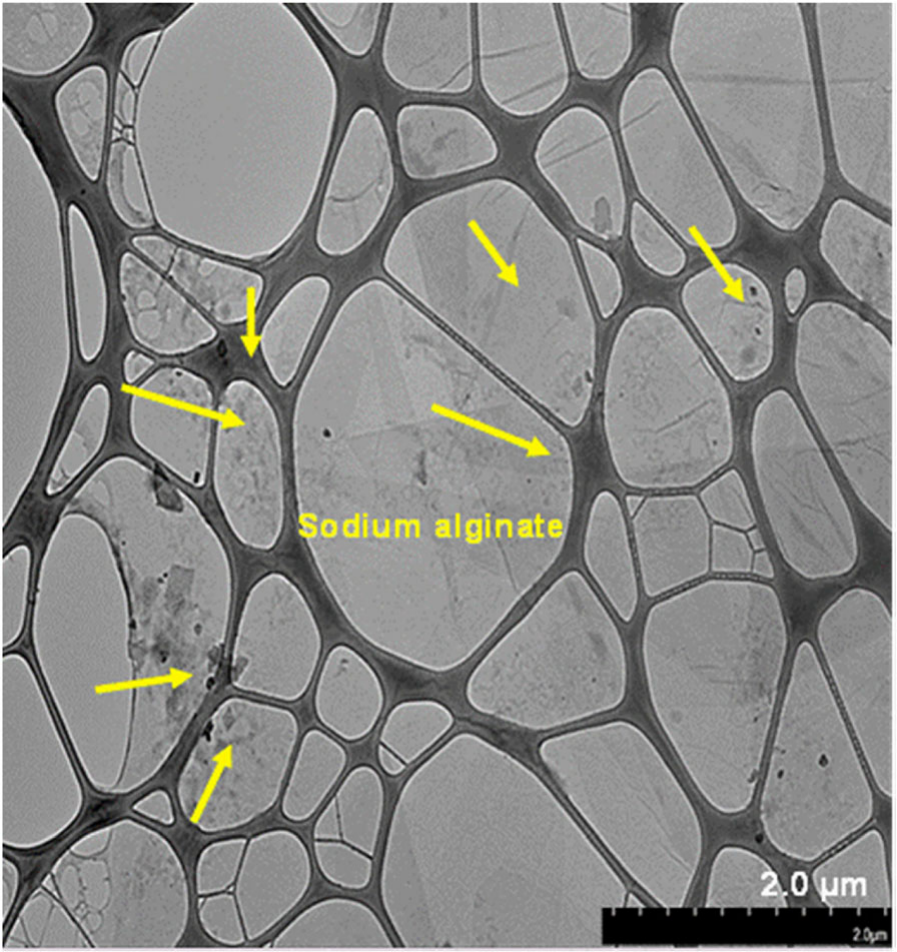
TEM image of SGO nanosheets distributed in sodium alginate polymer matrix

The surface image and cross section of the SA and SA/SGO bio membranes are shown in Fig. 4. Figure 4a–c is a surface image, and Fig. 4d–f is a cross-sectional image of membranes with different SGO contents. Both low and high enlargements show that the SGO sheet is completely dispersed homogeneously in the overall polymer matrix and is guided by intermolecular interactions; it is recognized that hydrogen bonds occur between the sulfonic acid groups in SGO and polar groups (-O-, C = O) in the SA/SGO membrane [45]. SGO is placed in the polymer matrix to function as a barrier to methanol molecules. The image for SA/SGO6 looks better with the full spread to the entire sodium alginate polymer matrix. Figure 5 is a TEM image for the composite formed in which the SGO nanosheets are well distributed in the sodium alginate polymer matrix. Sodium alginate exists in the nanosphere particle structure, which is similar to the previous study reported by Marrella et al. [46].

**Fig. 6:**
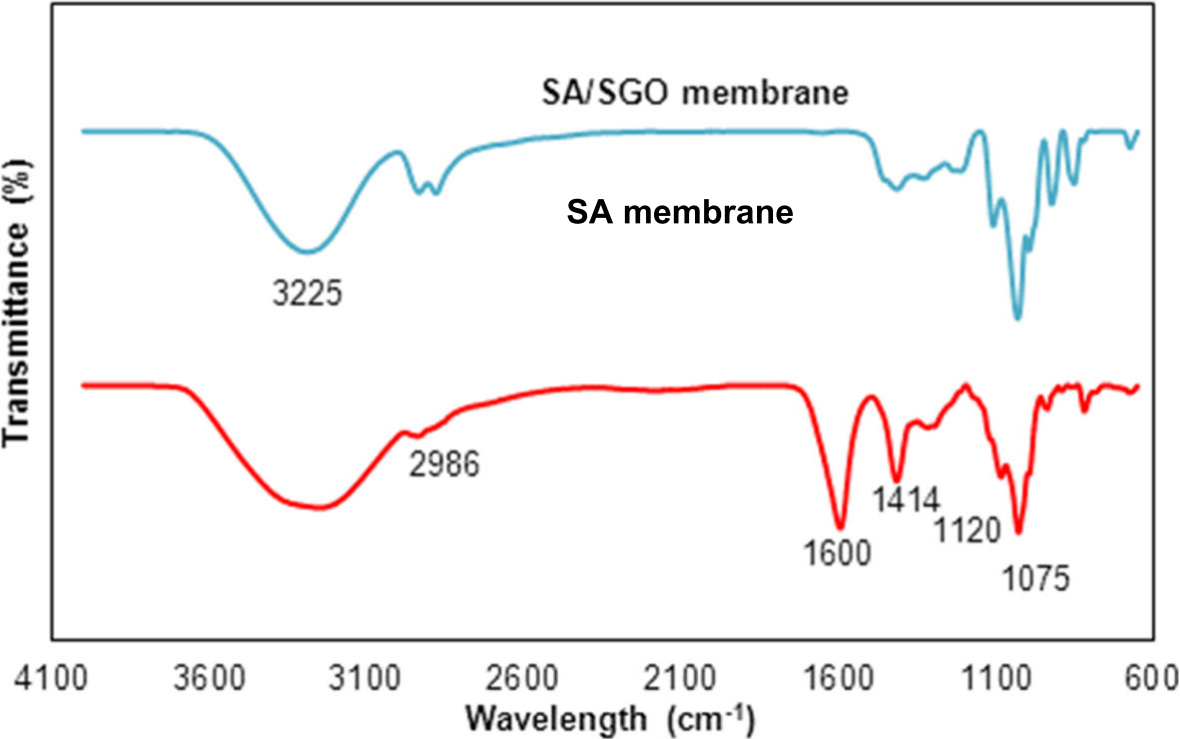
FTIR spectra of SA and SA/SGO membrane

The presence of hydrogen bonding interactions between SGO and the alginate polymer matrix is shown by the FTIR analysis. The FTIR results for the alginate and SGO alginate membranes are shown in Fig. 6. A slight shift seems to occur for the hydrogen bonding site spectra according to the hydrogen bond interactions. The O-H group bands in the alginate membrane appeared at 1413 and 3440 cm^−1^; however, the bands were shifted to 1406 and 3404 cm^−1^ in the SA/SGO membrane due to the hydrogen bonding among the polar groups in SGO and the O–H groups in alginate [45]. The C=O group bands in the alginate membrane also shifted to 1046 from 1082 cm^−1^. The location of the sulfonic group (–SO_3_H) bands in the alginate membrane also changed from 1284 to 1277 cm^−1^. Thus, the results show that there is hydrogen bonding between the SGO and alginate [47]. A complete dispersion of the SGO particles throughout the polymer matrix can facilitate the proton conduction path in all directions of the membrane. As a result, the properties of SA/SGO membranes were assumed superior to those of the pristine alginate membranes according to the SEM interior structure and the FTIR spectra.

### Thermal Stability and Mechanical Properties

**Fig. 7:**
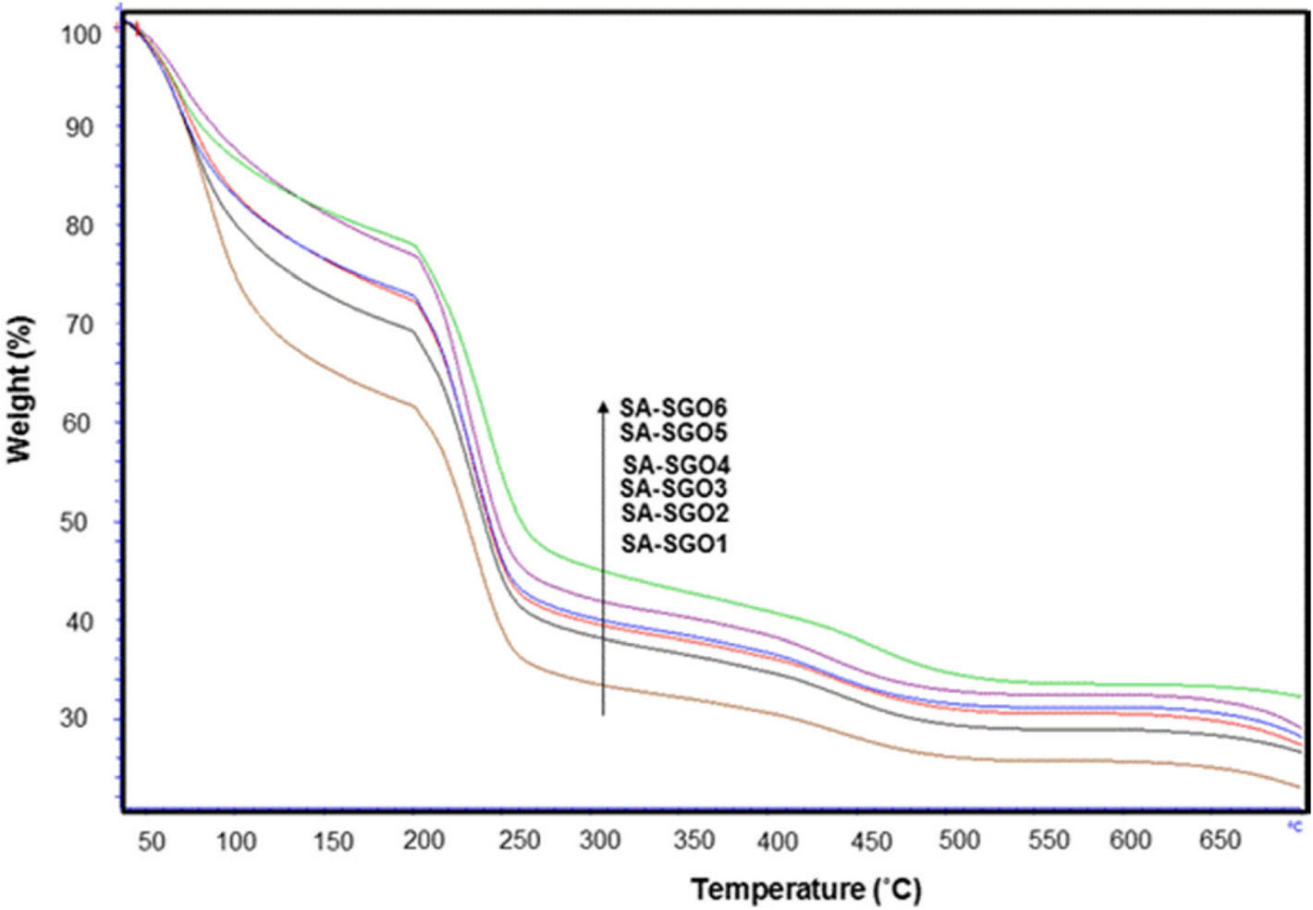
TGA curve for SA/SGO biomembranes with various SGO wt%

Figure 7 shows the comparison of TGA analysis for all SA/SGO biomembranes with different contents of SGO. Losses at the first stage occurred below 200 °C due to the release of water molecules, which is known as the evaporation process. Generally, thermal decomposition of GO is at a temperature of approximately 200 °C due to the decomposition of the oxygen labile group, while for alginate polymers, heat decomposition at the first stage is at 178 °C [48, 49]. The SA/SGO biomembrane shows a heavy loss at a higher temperature of 198 °C. This increased temperature indicates that there is an interaction between sodium alginate and SGO, which increases the heat resistance for SA/SGO biomembrane. This shows that the presence of SGO has increased the thermal stability of the biomembrane due to favorable interfacial interactions, such as hydrogen-bonding or electrostatic interactions between the sodium alginate matrix and sulfonated graphene oxide nanosheets, thus making this membrane fit for DMFC application. The second stage of weight losses occurs at a temperature of 250 °C due to the decomposition of the sodium alginate side-chain. The third stage (> 400 °C) involves the process of decomposition of the polymer backbone [50].

**Fig. 8:**
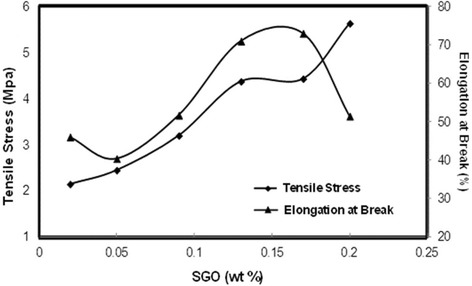
Tensile stress and elongation at break of biomembrane with various SGO wt%

Figure 8 presents the tensile stress and elongation at break of the membrane as the wt% of SGO varied. From 0.02 to 0.13 wt% of SGO, the tensile stress increased and then slightly decreased at 0.17 wt%. This might be attributed to the restacking of graphene oxide sheets, which can be related to the van der Waals forces in the GO nanosheets. The bulk of graphene oxide nanosheets leads to sliding and reduces the effect of graphene oxide in improving the mechanical properties of the membrane. The tensile stresses of Nafion and other biomembranes in previous studies are listed in Table 1 [51–55]. The Nafion membrane has a higher tensile stress compared to the SA/SGO6 biomembrane. However, it is comparable between biomembrane categories. The graphene oxide itself has very good mechanical properties, with an elastic modulus of 1100 GPa and an intrinsic strength of 125 GPa; this is the primary reason why SGO can increase the mechanical properties of the alginate membrane [45].

**Table 1:** Thickness, IEC, proton conductivity, methanol permeability, and membrane selectivity of SA/SGO composite biomembrane with different SGO content

Sample	Thickness (μm)	IEC (meq g^−1^)	Proton conductivity (σ, mS cm^−1^)	Methanol permeability (P, × 10^−7^ cm^2^ s^−1^)	Selectivity (SP × 10^4^ S s cm^−3^)	Reference
SA	198 ± 1	0.25 ± 0.03	6.36 ± 0.1	1.687 ± 0.22	3.7678	Current study
SA/SGO1	200 ± 1	0.31 ± 0.05	6.63 ± 0.4	2.657 ± 0.39	2.495	Current study
SA/SGO2	201 ± 2	0.34 ± 0.03	7.18 ± 0.5	2.453 ± 0.27	2.927	Current study
SA/SGO3	203 ± 1	0.38 ± 0.02	7.75 ± 0.2	2.351 ± 0.23	3.296	Current study
SA/SGO4	200 ± 1	0.45 ± 0.05	9.30 ± 0.1	2.045 ± 0.25	4.547	Current study
SA/SGO5	201 ± 3	0.48 ± 0.03	10.6 ± 0.1	1.738 ± 0.23	6.098	Current study
SA/SGO6	205 ± 2	0.56 ± 0.05	13.2 ± 0.1	1.535 ± 0.24	8.555	Current study
Nafion 117	–	0.86	0.098	12.3	7.967	[28]
Nafion 117	–	–	0.081	20	4.05	[29]
Nafion 117	–	–	0.1056	25	4.22	[30]

Moreover, the formation of hydrogen bonds between SGO and the pure alginate matrix polymer can also result in good mechanical properties. A greater formation of hydrogen bonding results in a stronger interfacial adhesion, consequently improving the mechanical strength of the membrane. The elongation at break pattern is in contrast to the tensile stress pattern. A lower tensile stress results in a higher elongation at break percentage. Elongation at break indicates to what extent the membrane film can be stretched until the maximum point, which is also known as flexibility. Table 1 compares several membranes from previous studies with the membrane of the current study in terms of elongation at break [51–56]. The different patterns between tensile stress and elongation at break are logical. As mentioned above, the presence of SGO in the membrane increases the interfacial linkage due to the hydrogen bonding, thus reducing the flexibility of the membrane.

### Liquid Uptake and Swelling Ratio of Membrane

**Fig. 9:**
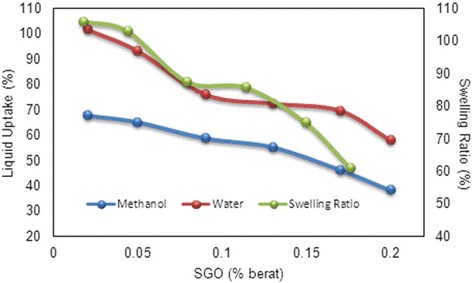
Liquid uptake and swelling ratio of SA/SGO membrane with wt.% of SGO

It is acknowledged that water is the prominent component in the proton exchange membrane because it acts as a proton conductor in which the adsorbed water facilitates proton transport [39]. Figure 9 presents the results of water uptake and methanol uptake of the SA/SGO membrane with varying SGO wt% values. As presented, the SA/SGO membrane has a lower water uptake capacity with different contents of sulfonated GO (lowest WU - 57.9% by SA/SGO6) in the membrane compared with pure alginate. An increasing amount of SGO reduces the water uptake due to its blocking ability as a filler in the membrane [5]. The addition of SGO facilitates the contraction of ionic pathways, thus hindering the movement of water and methanol. A higher SGO content results in a stronger barrier for the water absorption of the membrane. The hydrogen bonding between the SGO filler and the sodium alginate polymer strengthens the interfacial adhesion of the membrane composite, thus reducing the water uptake capacity [19]. The hydrogen bonding formation in the SA/SGO membrane involves the –OH groups in GO, the –O- and C=O groups on the SA chains, and contributions by sulfonate groups (–SO_3_H) [3, 19]. Similar to the pattern of the water uptake result, the methanol uptake of the SA/SGO membrane also decreased with increasing SGO wt% in the membrane. The presence of the same trend shows that there was good networking and bonding between SGO and the alginate polymer, which impeded fuel crossing. From the experimental result, the presence of graphene oxide-based materials lowered the water uptake capacity of the SA membrane and maintained its mechanical strength. The swelling ratio decreased from 106% to 61.12% with increasing SGO wt% in the alginate polymer matrix (Fig. 9) due to the blocking effect [10]. The strong hydrogen bonding also diminished the pathways for absorbance of the ionic group into the polymer [32].

### IEC, Proton Conductivity, Methanol Permeability, and Selectivity

Ion exchange capacity (IEC) calculation is important since it is responsible for measuring the number of milliequivalents ions in 1 g of the prepared membranes and is an indicator for proton conductivity in DMFCs.

**Fig. 10:**
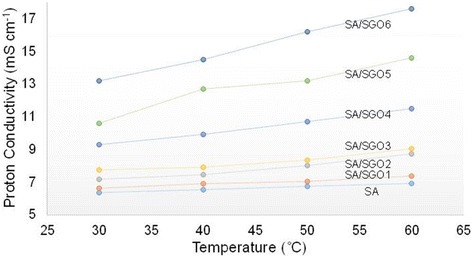
Proton conductivity of SA/SGO biomembranes with various content of SGO at different temperature

**Fig. 11:**
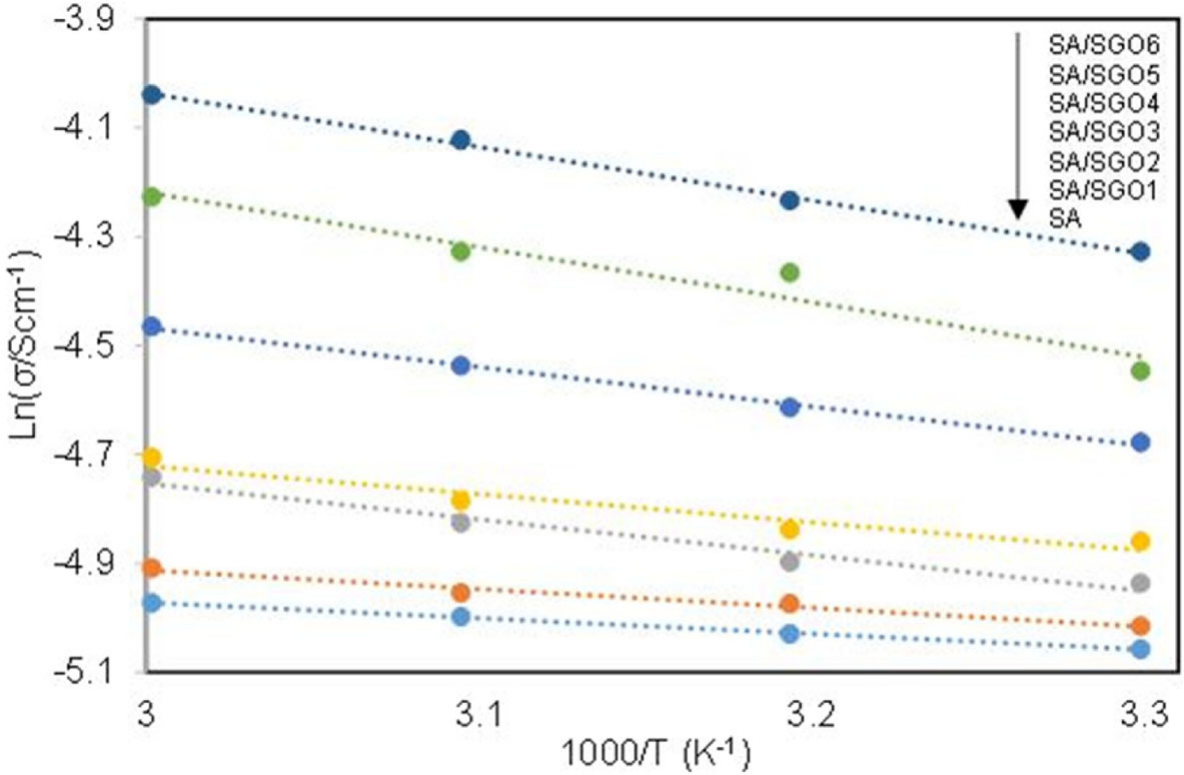
ln σ vs. 1000/T plot for the cross-linked QAPVA membranes, the lines indicate the linear regression

Table 2 shows the IEC values of the membranes. A higher IEC value is achieved by the SA/SGO membrane containing a higher wt% of SGO. This is due to the function of sulfonic acid groups in the SGO nanosheets. An increment in the IEC value increases the proton conductivity value of the SA/SGO biomembrane. The proton conductivities of the SA/SGO membrane versus temperature are presented in Fig. 10. Increasing the temperature leads to the enhancement of proton conductivity. The SA/SGO membrane features a consistently increasing pattern in proton conductivity as the SGO particle amount increases, with a maximal value of 13.2 mS cm^−1^ at 0.2 wt% of SGO loading at temperature of 30 °C. The ln *σ* vs. 1000/*T* plot is also shown in Fig. 11. Assuming that the conductivity follows an Arrhenius behavior, the ion transport activation energy *E*_a_ of the SA/SGO membranes can be obtained according to the Arrhenius equation:

$$ {E}_a=-b\ x\ R $$

where *b* is the slope of the line regression of ln *σ* (S/cm) vs. 1000/*T* (K^−1^) plots, and *R* is the gas constant (8.314472 JK^−1^ mol^−1^). The ion transport activation energy of the SA/SGO6 composite membrane is 8.17 kJ mol^−1^, which is slightly greater than the *E*_a_ of Nafion® 115 (6.00 kJ mol^−1^) [57] and lower than that of Nafion 117 (12 kJ mol^−1^) [58]. This can be attributed to the hydrophilic properties of the sodium alginate matrix, which provide high water content, and the introduction of SGO still allows this property to remain due to the hydrophilic properties of oxygenated functional groups. The abundant water forms a continuous transferring channel and makes the movement of ion easy.

**Table 2:** Comparison of condition in single-cell performance test with power density result for previous work and current study

Membrane	Anode catalyst loading (mg cm^−2^)	Cathode catalyst loading (mg cm^−2^)	Methanol feed concentration (mol dm^−3^)	Temperature (°C)	Pmax (mW cm^−2^)	Mode	Reference
SA	Pt-Ru:8	Pt:8	4	RT	2.8	Passive	Current study
SA/SGO6	Pt-Ru:8	Pt:8	4	RT	5.9	Passive	Current study
Nafion 117	Pt-Ru:8	Pt:8	4	RT	6.6	Passive	Current study
Nafion 117	Pt-Ru:8	Pt:8	2	RT	7.2	Passive	[28]
Alginate-carrageenan	Pt-Ru:5	Pt:5	2	50	10.4	Active	[31]

Figure 13a presents the suggested proton mobility mechanism in SA/SGO plasticized with glycerol in which high synchronization exists between H^+^ and electron lone pairs belonging to the oxygen atoms carrier in glycerol and the hydrophilic sulfonic acid groups in SGO nanosheets. We believe that the proton transport applies both Grotthus and vehicle mechanisms, strengthened by the SGO particles.

**Fig. 12:**
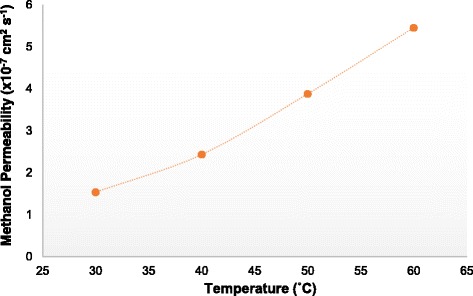
Methanol permeability of membrane SA/SGO6 vs. temperature

The SA/SGO biomembranes show very low methanol permeability, and the lowest was achieved by SA/SGO6 (1.535 × 10^−7^ cm^2^ s^−1^), as listed in Table 2. The low methanol permeability can be explained in terms of the membrane microstructure between sodium alginate, SGO, and glycerol plasticizer. The introduced SGO particles serving as fillers in the SA polymer create substantial obstacles to the linked hydrophilic passages. The SGO filler blocks the migration of methanol passing through the membrane, and this is known as the blocking effect, which reduces the methanol permeability. The methanol permeability also decreases because of the interfacial interaction between the SGO and SA biopolymer [41]. The methanol permeability of the SA/SGO6 bio membrane at four different temperature conditions is shown in Fig. 12. As seen, the methanol permeability increases at a higher temperature, which can be related to the structure changes of the bio membrane. The higher temperature provides more heat, which can shake the membrane chains and molecules, thus leading to more free volume, which consequently reduces the methanol blocking effect. Less resistance causes easier movement of methanol diffusion [59]. Mu et al. [60] reported the decrease in methanol crossover in the presence of Au nanoparticles self-assembled on a Nafion membrane, which consequently improved the overall performance.

**Fig. 13:**
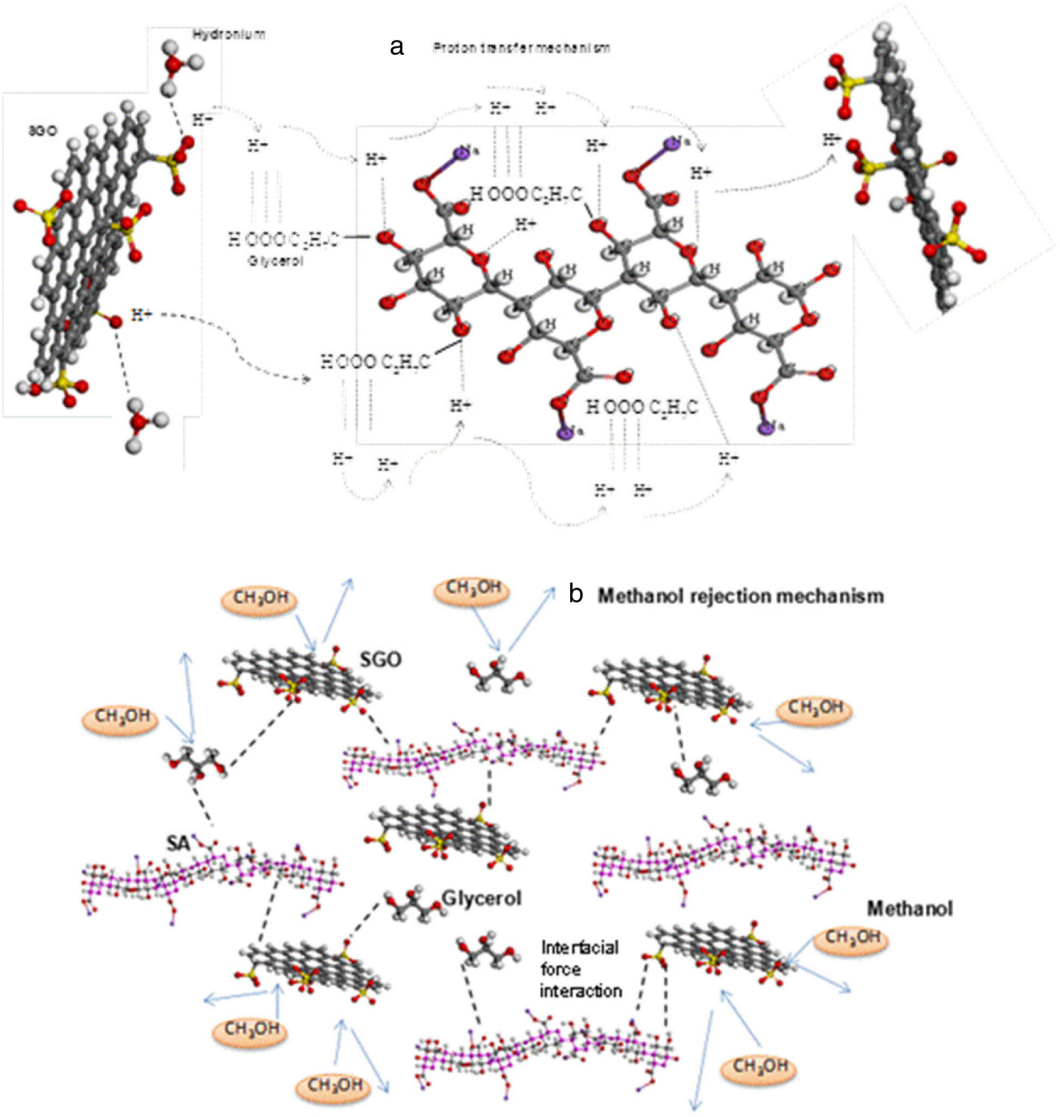
Suggested mechanism of **a** proton mobility and **b** methanol rejection

The interfacial interaction between SGO filler, glycerol, and SA polymer confines the hydrophilic passage formation in the membrane, and this wide hydrophilic passage is a significant factor in methanol migration [19]. Thus, the presence of SGO facilitates methanol permeability reduction [6]. The proposed mechanism of methanol rejection is presented in Fig. 13b.

It was noticed that a higher selectivity value resulted in a higher DMFC capability. The selectivity values of the SA/SGO can be observed in Table 2, which compares the selectivity among SA and SA/SGO biomembranes as well as Nafion 117 membranes from previous work. The presence of SGO enhanced the selectivity of the SA/SGO polymer membrane (8.555 × 10^4^ S s cm^−3^ for 0.2 wt% SGO loading), which is higher than that of SA (3.7678 × 10^4^ S s cm^−3^) and fortunately also higher than that of Nafion 117 (7.99 × 10^4^ S s cm^−3^) [38], 4.05 × 10^4^ S s cm^−3^ [61], and 4.22 × 10^4^ S s cm^−3^ [62], in which the low methanol permeability is the main factor to be considered.

### Single Cell

#### Single-Cell Performance Evaluation

**Fig. 14:**
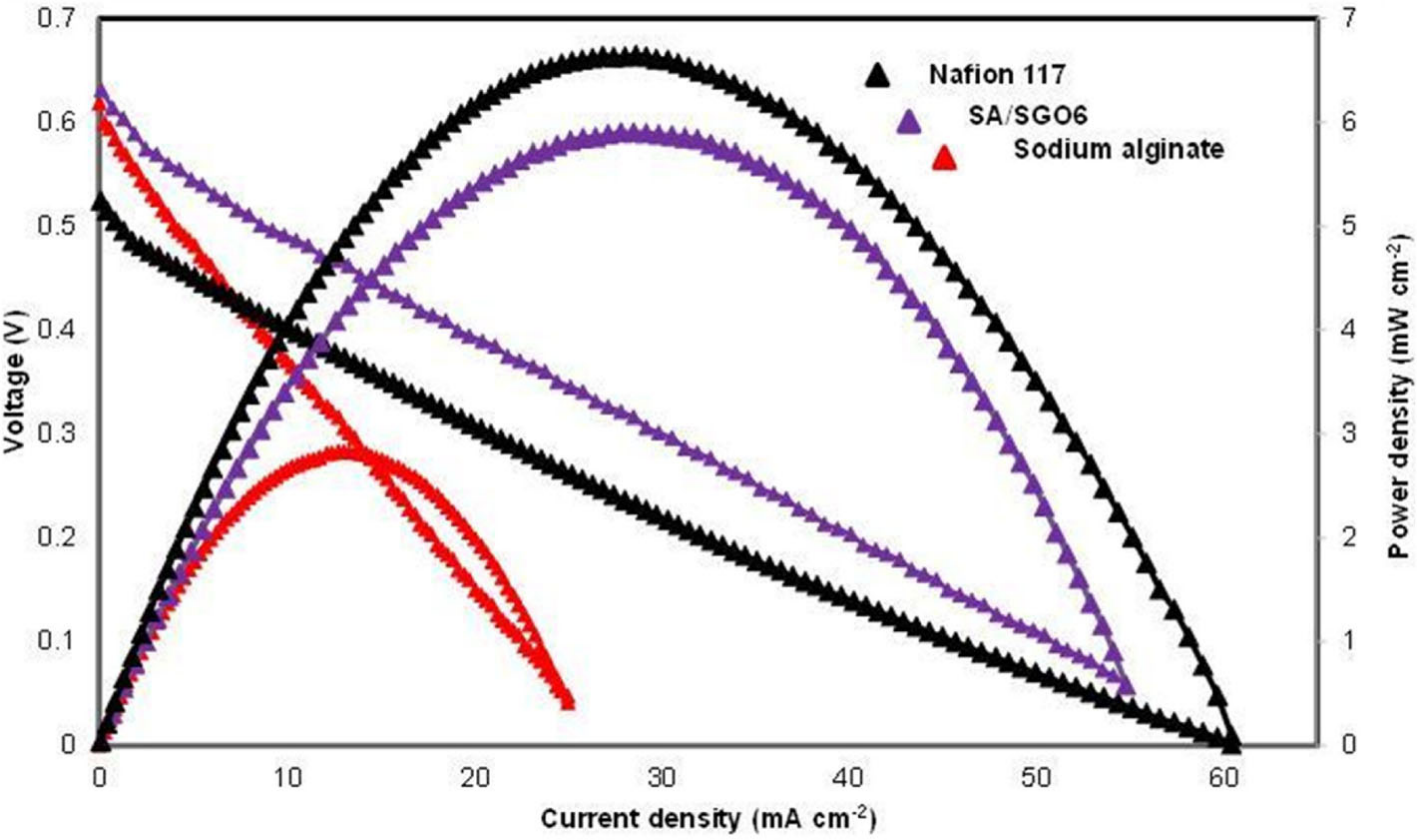
Single-cell performance test for sodium alginate, SA/SGO6, and Nafion 117 (4 M methanol and 25 °C temperature, passive mode)

Figure 14 indicates the cell polarization result for pure alginate, SA/SGO6 composite biomembrane and Nafion 117 under ambient temperature, 4 M methanol concentration and passive mode condition. The SA/SGO6 composite biomembrane was applied due to the high selectivity factor and obviously had a higher open-circuit voltage (0.63 V), which can be related to the low methanol permeability equaling to that the sodium alginate biomembrane. The OCV of Nafion 117 (0.52 V) in the current study is lower than SA/SGO and sodium alginate, which might be due its higher methanol permeability. The crossing of methanol through the membrane leads to the reduction in the OCV value. The higher OCV of SA/SGO and alginate membrane is the big indicator that synthesized membrane has lower methanol permeability compared to Nafion, which the main objective of this study is successfully achieved. The improvement in the power density of SA/SGO6 is due to the sulfonic acid group that functions as a proton transferral pathway as well as a methanol inhibitor, thus achieving 5.9 mW cm^−2^ compared to the sodium alginate, which achieved only 2.83 mW cm^−2^. However, Nafion 117 achieved a higher power density, which was 6.62 mW cm^−2^. Thiam et al. [38] reported the performance of Nafion 117 membrane under the same condition with a power density of 7.95 mW cm^−2^. No doubt, Nafion achieves a better performance in DMFC application due to the excellent proton conduction. However, the power density performance between Nafion 117 and SA/SGO biomembranes does not show a big difference quantitatively. Hence, SA/SGO can be an alternative membrane for DMFC in the future. However, the properties of the membrane still need to be enhanced, and higher wt% of SGO filler can probably be used to obtain a higher power density. To the best of our knowledge, there is only one previous work by Pasini Cabello et al. that has examined the single-cell performance in DMFC application using an alginate biopolymer-based membrane [18]. They tested an alginate/carrageenan membrane at temperatures of 50, 70, and 90 °C in 2 M methanol concentration in the active mode, which achieved maximum power densities of 10.4, 13.9, and 17.3 mW/m^2^, respectively. The active mode has an advantage due to the continuous flow of the methanol feed into the cell that allows the reaction to occur continuously and thus is capable of achieving a higher power density. The higher power density could be achieved at a higher temperature due to the higher number of activated protons. Nevertheless, this work is an indicator that biopolymer-based membrane has a big potential that can be explored and applied in DMFC systems.

## Conclusions

In conclusion, a membrane with low methanol permeability, high proton conductivity, and high selectivity was successfully prepared through the simple technique known as the blending method. The presence of sulfonated graphene oxide enhanced the properties of the alginate-based polymer membrane in terms of proton conductivity and methanol permeability. The sulfonate groups facilitated the networking between the alginate polymer and the graphene oxide filler. The blocking effect of SGO also reduced the methanol crossover in the membrane. The primary weaknesses of the alginate polymer, which are its mechanical properties of tensile strength and elongation at break, were also improved by the addition of SGO into the polymer matrix. The presence of SGO improved the SA/SGO membrane to a high level comparable to commercial membranes.

## Abbreviations

BC: Bacterial cellulose
CNC: Cellulose nanocrystal
CNFs: Cellulose nanofibers
CNT: Carbon nanotube
DI: Deionized
DLFC: Direct liquid fuel cell
DMFC: Direct methanol fuel cell
EDX: Energy dispersive X-ray
FESEM: Field emission scanning electron microscope
FTIR: Fourier transform infrared
GO: Graphene oxide
GOS: Graphene oxide sheet
HRTEM: High-resolution transmission electron microscopy
IEC: Ion exchange capacity
*L*: Distance between the two electrodes
OCV: Open circuit voltage
P: Membrane diffusion permeability for methanol
PEMFC: Polymer electrolyte membrane fuel cell
PEMs: Proton exchange membrane
PMA: Phospho molybdic acid
PSSA: Poly-styrene sulfonic acid
PVA: Poly vinyl alcohol
PVP: Poly (vinyl pyrrolidone)
*R*: Resistance of the membrane
RGO: Reduced graphene oxide
SA: Sodium alginate
SA/SGO: Sodium alginate/sulfonated graphene oxide membrane
SGO: Sulfonated graphene oxide
SHNT: Sulfonated halloysite nanotube
SPSF: Sulfonated polysulfone
SW%: Swelling ratio percentage
*T*: Membrane thickness
TGA: Thermal gravimetric analysis
*W*: Width of the membrane
WU%: Water uptake percentage
XPS: X-ray photoelectron spectroscopy
